# Liberal Versus Restrictive Transfusion Trigger after Acute Brain Injury: More than Just Higher Blood Oxygen Content

**DOI:** 10.1089/neur.2025.0008

**Published:** 2025-03-19

**Authors:** Adrienne K. Ho

**Affiliations:** Department of Public Health Sciences, Queen’s University, Kingston, Ontario, Canada.

**Keywords:** brain injury, transfusion

## Abstract

Previous studies on restrictive versus liberal blood hemoglobin transfusion triggers in critically ill or cardiac surgery patients had established a paradigm favoring restrictive triggers. In contrast, the hemoglobin transfusion threshold in traumatic brain injury optimization and transfusion strategy in patients with acute brain injury trials (2024) suggest that a liberal hemoglobin (90–100 g/L) transfusion trigger is associated with better neuro-outcomes than a restrictive (70 g/L) trigger in anemic patients with acute brain injury. Increased blood oxygen content is one obvious possible reason for the observed superior neuro-outcomes with more liberal red blood cell transfusion. In this author’s opinion, another plausible reason is that in replacing extracranial blood loss, which most of the patients in both trials had, avoidance of blood alternatives that could worsen intracranial hypertension might also have benefitted the liberal transfusion cohorts.

Anemia is associated with reduced blood oxygen-carrying capacity. While red blood cell (RBC) transfusion can improve the oxygen content in blood, it can cause adverse effects. Thus, whether anemic vulnerable patients should receive RBC transfusion has always been of great interest to clinicians.^1–7^

There were two important multicenter randomized controlled trials (RCTs) published in 2024 on adults with acute brain injury (ABI) with concomitant anemia, comparing restrictive vs. liberal transfusion thresholds based on a blood hemoglobin of ≤70 g/L versus ≤100 g/L (HEMOTION trial: hemoglobin transfusion threshold in traumatic brain injury optimization^1^) and based on a hemoglobin of <70 g/L vs. <90 g/L (TRAIN trial: Transfusion Strategy in Patients with ABI^2^). Patients were screened by investigators on admission to the intensive care unit (ICU) in both studies, and those eligible were randomized. The thresholds were maintained for the full duration of the ICU stay, which was 15 (mean) days in the HEMOTION trial and ∼22 days in the TRAIN trial.

In HEMOTION, all subjects had been involved in serious trauma such as vehicle accidents, assaults, falls, etc. On entering the trial, their mean lowest hemoglobin level was ∼84 g/L. Hemoglobin level was measured at least once daily, whenever arterial blood gases were measured. For the entire duration of their ICU stay, patients in the liberal group received RBCs to achieve a blood hemoglobin of ≥100 g/L, whereas patients in the restrictive group only received RBCs if the blood hemoglobin dropped to ≤70 g/L. An unfavorable 6-month Glasgow Outcome-Scale-Extended (GOS-E) score occurred in 263/358 (73.5%) in the restrictive group and in 249/364 patients (68.4%) in the liberal group (higher risk for restrictive: adjusted absolute risk difference +5.4%, 95% confidence interval [CI]: −2.9 to 13.7).

In TRAIN, 60.5% of the subjects had trauma-related brain injury; the rest had aneurysmal subarachnoid (21.7%) or intracerebral (17.9%) hemorrhage. The subjects (age 52 ± 16 years) had more chronic disease than those in the HEMOTION trial (age 48.9 ± 18.8 years). The mean baseline lowest hemoglobin was ∼84 g/L on day 1. Apart from a slightly different upper/liberal threshold, the transfusion strategy was similar to that used in HEMOTION. An unfavorable 6-month GOS-E score occurred in 300/413 (72.6%) in the restrictive group and in 246/393 (62.6%) in the liberal group (higher risk for restrictive: absolute risk difference +10%, 95% CI: 3 to 16).

There have been previous multicenter RCTs comparing restrictive and liberal transfusion strategies in anemic adult patients who were critically ill,^3^ in septic shock,^4^ with acute myocardial infarction,^5,6^ and undergoing cardiac surgery.^7^ ABI was not within the scopes of those studies, only one of which included a very small number of patients (<0.05%) with unspecified neurological abnormalities;^3^ hence, the need for studies such as HEMOTION and TRAIN. All of the studies used similar blood hemoglobin thresholds and compared mortality,^3–7^ major adverse cardiovascular events,^3–7^ thromboembolic stroke,^4,7^ and renal failure^4,7^ rates. They had shown that a restrictive strategy is superior,^3^ similar,^4^ or non-inferior^5–7^ to a liberal strategy. Together, they underpin the prevailing “less is more” paradigm of avoiding the unnecessary use of a valuable resource that is not without complications unless the blood hemoglobin drops below 70–80 g/L.

In contrast, both the HEMOTION and TRAIN studies suggest a restrictive erythrocyte transfusion strategy is inferior^2^ or possibly inferior.^1^ What makes HEMOTION and TRAIN different? Improved blood oxygen carrying capacity resulting in higher oxygen delivery to an injured and congested brain is a clear and logical reason. In this author’s view, in the setting of raised intracranial pressure (ICP), a compromised blood–brain barrier, and disturbed cerebral autoregulation, a restrictive erythrocyte transfusion strategy is intrinsically inferior for possibly another important reason, one that was not discussed by the authors of these two trials^1,2^ and of the accompanying TRAIN editorial.^8^

In ABI, blood loss within the cranium is insufficient to cause a significant drop in hemoglobin, and extracranial injuries are the main cause of any associated acute anemia.^9^ Not surprisingly, more than two-thirds of the HEMOTION cohort had extracranial injuries, and only <1% of HEMOTION patients suffered from chronic anemia before their injuries. The same can probably be said about the 58.2% of the TRAIN restrictive cohort that had trauma-related ABI. Likewise, subarachnoid and intracerebral hemorrhages do not acutely cause anemia, and one must invoke preexisting anemia, comorbidities, and, most importantly, extracranial bleeding in those subsets within the TRAIN cohort.

One may therefore logically argue that virtually all the HEMOTION patients and the majority of the TRAIN restrictive patients had lost enough blood outside the brain to cause severe anemia and therefore required substantial volume replacement to prevent hypoperfusion of the brain and other organs. Indeed, in HEMOTION, 83 (22.7%) of the 366 patients in the liberal group and 105 (28.8%) of the 364 patients in the restrictive group were hypotensive on admission to ICU. In the liberal groups, RBC transfusion was given. In the restrictive cohorts, if RBC transfusion was disallowed until hemoglobin dropped to ≤70 g/L, what were the fluids given instead, and could such fluids have contributed to the poorer neuro-outcome in those cohorts?

For volume replacement, alternatives to RBC transfusion include crystalloids and colloids. Unlike RBCs, which remain intravascular after transfusion, infused crystalloid solutions quickly leave the intravascular space such that 3–4 times the volume of Ringer’s lactate or normal saline is required for each volume of RBC transfused.^10^ In HEMOTION, the mean hemoglobin difference between the restrictive and liberal cohorts was ∼20 g/L throughout the 15 (median)-day ICU stay. Patients in the restrictive cohort received an average of 0.8 (interquartile range [IQR] 0–1) unit of RBCs/person during their ICU stay, whereas those in the liberal cohort received an average of 4 (IQR 2–5) units of RBC/person, resulting in an average difference of roughly 3–4 units/patient. Based on the IQRs, the difference was substantially higher in many patients. Packed RBCs come in 200–400 mL/unit. For a median of 300 mL/unit, 3–4 units of packed RBCs *in vivo* require the administration of ≥3–4L of crystalloid. The same argument can be applied to the TRAIN trial, in which the mean hemoglobin difference between the two cohorts was ∼15 g/L throughout the ∼22-day ICU stay. Crystalloids potentially exacerbate brain swelling, especially when the blood–brain barrier and cerebral pressure autoregulation have been compromised, an assumption that might be applied to all of the patients in both trials. Furthermore, the commonly used crystalloid solutions, Ringer’s lactate and Plasma-Lyte, are slightly hypo-osmolar (273 and 294 mOsmol/L, respectively), which may further exacerbate raised ICP. Use of Plasma-Lyte or Ringer’s lactate was associated with worse discharge disposition compared to normal saline in patients with traumatic brain injury.^11^ Normal saline, with its high chloride content, can contribute to metabolic acidosis, coagulopathy, and renal injury when given in high volume.^12^ Based on results from the Saline or albumin for fluid resuscitation in patients with traumatic brain injury (SAFE) trial, albumin is associated with a worse neurological outcome than normal saline in critically ill patients with traumatic brain injury.^13^ Synthetic colloids are rarely used because of associated negative effects on coagulation and kidney functions and may adversely impact survival in TBI.^14^ In the liberal groups, RBC transfusion avoids many of the aforementioned problems of non-blood volume expanders. Indeed, in the TRAIN study, salvage therapy for raised ICP was required in 110 patients in the liberal cohort of 397 (27.7%) and in 135 patients in the restrictive cohort of 423 (31.9%) (*p* = 0.19). Correspondingly, cerebral ischemia occurred in 35 (8.8%) patients in the liberal group and 57 (13.5%) patients in the restrictive group (relative risk lower in the liberal transfusion cohort: 0.65, 95% CI 0.44 to 0.97; *p* = 0.03).

In animal cerebral injury models, normovolemic anemia increased ICP,^15^ impaired cerebrovascular CO_2_ responsiveness,^16^ and worsened neurooutcome.^17^ In a mathematical model of anemia in focal stroke in rabbits, oxygen uptake by ischemic penumbra decreased progressively when the hemoglobin level decreased to <100 g/L (normal range: 98–158 for females, 104–174 g/L for males). In other words, there is no need to put the unit for males as it is understood that the unit is g/L.^18^

Brain perfusion dynamics are unique. Confined within the skull and having lost its natural defences, the injured swelling brain needs not just blood with enough oxygen-carrying capacity but, more critical than any other organs, also avoidance of congestion to ensure adequate perfusion. HEMOTION and TRAIN suggest that ABI does not belong to the “less is more” paradigm for RBC transfusion previously established in critical patients^3–6^ and those undergoing cardiac surgery.^7^ In this author’s opinion, the benefits of RBC transfusion in patients with ABI who have concomitantly sustained substantial extracranial blood loss lie not necessarily entirely in increased blood oxygen carrying capacity but also in the superiority of RBC over crystalloid and colloid solutions in avoiding exacerbation of raised ICP. Could it be possible that, to avoid crystalloid, albumin, and synthetic colloids, an even more liberal hemoglobin target (e.g., 120 g/L) would be more beneficial in patients with ABI who happen to require additional blood volume replacement? Modern blood products are very safe. Indeed, consequential adverse outcomes from transfusion were similar between the restrictive and liberal cohorts in both the HEMOTION and TRAIN studies. Furthermore, whereas there is evidence that crystalloids, albumin, and colloids may cause further harm in ABI, there is no evidence that RBCs *per se* exacerbate ABI, which brings us back to the same question: If a hemoglobin of 90–100 g/L is better than 70 g/L, why not an even higher target of, say, 120 g/L.

HEMOTION and TRAIN are two landmark trials that have shown that in ABI more RBCs may be better. Further studies are required to determine how much more, as well as when and why.

## Transparency, Rigor, and Reproducibility Statement

This commentary involves no original data. It is not a prospective or retrospective study on human or nonhuman subjects. It analyzes two major randomized controlled trials and attempts to introduce a perspective that has apparently not been considered by the investigators of those studies.

## Abbreviations Used

GOS-E: Glasgow Outcome-Scale-Extended
ICP: intracranial pressure
RBC: red blood cell
RCTs: randomized controlled trials
